# Argonaute with stepwise endonuclease activity promotes specific and multiplex nucleic acid detection

**DOI:** 10.1186/s40643-021-00401-6

**Published:** 2021-06-11

**Authors:** Guanhua Xun, Qian Liu, Yuesheng Chong, Xiang Guo, Zhonglei Li, Yinhua Li, He Fei, Kai Li, Yan Feng

**Affiliations:** 1 State Key Laboratory of Microbial Metabolism, School of Life Sciences and Biotechnology, Shanghai Jiao Tong University, Shanghai, 200240 People’s Republic of China; 2 Department of Obstetrics and Gynaecology, The Fifth People’s Hospital of Shanghai Affiliated To Fudan University, Shanghai, 200240 People’s Republic of China; 3 GeneTalks Biotechnology Inc., Changsha, 410013 Hunan China

**Keywords:** Thermophiles, Argonaute, Nuclease, Multiplex, Nucleic acid detection

## Abstract

Argonaute proteins (Agos) from thermophiles function as endonucleases via guide-target base-pairing cleavage for host defense. Since guides play a key role in regulating the catalytic specificity of Agos, elucidating its underlying molecular mechanisms would promote the application of Agos in the medical sciences. Here, we reveal that an Ago from *Pyrococcus furiosus* (*Pf*Ago) showed a stepwise endonuclease activity, which was demonstrated through a double-stranded DNA cleavage directed by a single guide DNA (gDNA) rather than a canonical pair of gDNAs. We validated that the cleavage products with 5'-phosphorylated ends can be used as a new guide to induce a new round of cleavage. Based on the reprogrammable capacity of Ago’s stepwise activity, we established a rapid and specific platform for unambiguous multiplex gene detection, termed Renewed-gDNA Assisted DNA cleavage by Argonaute (RADAR). Combined with a pre-amplification step, RADAR achieved sensitivity at the femtomolar level and specificity with at least a di-nucleotide resolution. Furthermore, RADAR simultaneously discriminated among multiple target sequences simply by corresponding multiple guides. We successfully distinguished four human papillomavirus serotypes from patient samples in a single reaction. Our technique, based on the unique properties of Ago, provides a versatile and sensitive method for molecular diagnosis.

**Supplementary Information:**

The online version contains supplementary material available at 10.1186/s40643-021-00401-6.

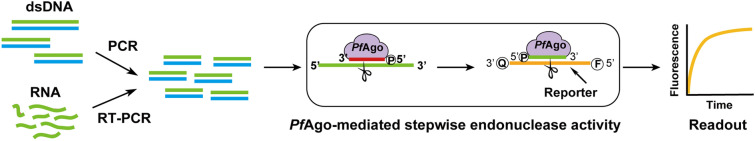

## Introduction

Argonaute proteins (Agos) play important roles in a wide range of biological processes, including gene regulation and host defense, by interacting with nucleic acid molecules (Hutvagner and Simard 2008; Makarova et al. 2009; Olovnikov et al. 2013; Swarts et al. 2014b). The role of Agos in molecule biology and its function as biomarker have also been investigated, in which eukaryotic Ago2’s activity can be accurately measured via the biosensor development (Zhang et al. 2018). Prokaryotic Agos are more diverse in their biochemical behavior than their eukaryotic counterparts. Thermophilic agents have attracted increasing interest because of their unique endonuclease activity as directed by guide DNA/RNA molecules (Swarts et al. 2015, 2014a; Willkomm et al. 2017; Yuan et al. 2005). An Ago from the hyperthermophilic archaeon *Pyrococcus furiosus* (*Pf*Ago) can perform precise DNA cleavage directed by small 5ʹ-phosphorylated single-stranded DNA (ssDNA) as guide DNA (gDNA) at 95 °C (Song et al. 2004; Swarts et al. 2015). The canonical cleavage product of *Pf*Ago was observed, with cleavage occurring at the opposite nucleotide 10/11 of the gDNA. Thermophilic Agos from *Thermus thermophilus* (*Tt*Ago) and *Methanocaldococcus jannaschii* (*Mj*Ago) showed similar endonuclease activity to *Pf*Ago, but differed in their substrate spectra or optimal temperatures (Swarts et al. 2014a; Zander et al. 2017). Moreover, double-stranded DNA (dsDNA) substrates can be cleaved upon prolonged incubation at high temperature under guide-free conditions (Swarts et al. 2017; Zander et al. 2017), which indicates that the ssDNA generated randomly via dsDNA instability might be used as a new guide and direct further DNA cleavage. Generally, the molecular mechanism by which guides regulate the binding and cleavage of the catalytic process remains unclear.

Presently, various PCR-based and isothermal amplification-derived nucleic acid detection methods have been well-established in the past few decades, but have trade-offs in sensitivity, specificity, and operational convenience (Milbury et al. 2009; Wang et al. 2020; Yan et al. 2014). Endonucleases can specifically recognize their target nucleic acids and are regarded as powerful tools to enhance the specificity and sensitivity of gene detection. Of particular note, the programmable endonuclease CRISPR system has recently revolutionized the field of diagnostics (Chen et al. 2018a; Gootenberg et al. , 2017, 2018; Li et al. 2018). Combined with amplification of PCR or RPA, CRISPR-based detection methods have been proposed for testing various targets, including pathogens and single nucleotide polymorphisms (SNPs). The main challenge of CRISPR is the restriction of PAM/PFS on the target sequence and the complexity of multiplexing for various targets being sensed (Wang et al. 2020). So far, only two multiplexed biosensing systems have been published based on CRISPR/Cas systems (Gootenberg et al. 2018; Wu et al. 2020). Therefore, the sensitive, rapid, and multiplex detection of nucleic acids is necessary for molecular diagnostic applications.

Classified as programmable endonucleases, thermophilic Agos were postulated to be suitable candidates for DNA manipulation applications (Enghiad and Zhao 2017; Hegge et al. 2018). Thermophilic Agos have been successfully exploited for rare SNV enrichment. Recently, *Tt*Ago was used to enrich SNV products after PCR amplification in a two-step operation called Nucleic Acid enrichment Via DNA Guided Argonaute from *Thermus thermophilus* (NAVIGATER) (Song et al. 2020). We applied the specific discriminated cleavage activity of *Pf*A to couple with PCR amplification in a single tube, named A-Star (Ago-directed Specific Target enrichment and detection). The special guide design enables the easy and efficient detection of rare mutations and achieves an over 5500-fold increase in the efficiency of detecting 0.01% rare mutations (Liu et al. 2021). For virus detection, He et al. developed a *Pf*Ago -mediated Nucleic acid Detection method (PAND) (He et al. 2019). However, it requires three individual gDNAs to stimulate cleavage and detect one target, but the cleavage mechanism is still not confirmed. Therefore, a deeper understanding of guide DNA-regulated target DNA cleavage is needed to provide an alternative approach for nucleic acid detection with a simple guide design and high sensitivity.

In this study, we first addressed the catalytic process of gDNA generation in the canonical DNA cleavage reaction. We observed that *Pf*Ago could cleave dsDNA substrates in a precise and regulated manner, guided by only a single gDNA instead of a pair of gDNA. Then, we characterized the single gDNA-directed stepwise dsDNA cleavage activity in detail via biochemistry analysis, such as mass spectrometry and binding shift assays. Subsequently, we developed a highly specific, multiplex detection platform that was used to distinguish four DNA targets in a convenient and efficient way, thereby demonstrating the utility of this Ago-based method for applications in biotechnology and molecular diagnostics.

## Materials and methods

### Protein expression and purification

A synthesized codon-optimized version of the *Pf*Ago gene was ordered from GenScript (Nanjing, China) and incorporated into the pET28a-derived plasmid pEX-*Pf*Ago with an N-terminal His-tag. This expression plasmid was transformed into *Escherichia coli* BL21(DE3) cells. A 5-mL seed culture was grown at 37 °C in LB medium with 50 μg/mL kanamycin, which was transferred to 1 L of LB medium in a shake flask containing 50 μg/mL kanamycin. The cultures were incubated at 37 °C until they reached OD_600_ values of 0.8–1.0. Protein expression was induced through the addition of isopropyl β-d-thiogalactopyranoside (IPTG) to a final concentration of 1 mM, followed by incubation for 16 h at 20 °C. Cells were harvested via centrifugation for 20 min at 6000 rpm and 4 °C, and the cell pellet was collected for further purification.

The cell pellets were resuspended in lysis buffer (20 mM Tris/HCl, 1 M NaCl, 2 mM MnCl_2_, pH 8.0) and then disrupted using a high-pressure homogenizer at 600–800 bar for 3 min (Gefran, Italy). The lysates were centrifuged for 30 min at 12,000 rpm and 4 °C, and the supernatants were used for Ni–NTA affinity purification with an elution buffer (20 mM Tris/HCl, 1 M NaCl, 2 mM MnCl_2_, 200 mM imidazole, pH 8.0). Further gel filtration purification (Superdex 200, GE Healthcare, USA) was carried out over a salt concentration of 1 M NaCl in an elution buffer (20 mM Tris/HCl, 1 mM DTT, 5% glycerol, 2 mM MnCl_2_, pH 8.0). The resulting fractions from gel filtration were analyzed via SDS-PAGE, and fractions containing the protein were flash frozen at − 80 °C in a storage buffer (20 mM Tris–HCl, pH 8.0, 250 mM NaCl, 0.5 mM MnCl_2_, 10% (v/v) glycerol).

### Nucleic acid preparation

The ssDNA target, gDNA, primers, and fluorophore quencher (FQ) reporters were synthesized commercially (Sangon Biotech, Shanghai, China). dsDNA targets 600-bp in length were synthesized by GenScript (Nanjing, China) in the form of pET-28a-derived plasmids. Plasmid DNA was extracted using a Plasmid DNA MiniPrep Kit (Generay, China). A 100-bp dsDNA target was obtained by amplifying the corresponding plasmids with primers designed using NCBI Primer-BLAST with the following parameters: amplicon size, between 90 and 120 bp; primer melting temperature, between 54 and 67 °C; and primer size, between 20 and 25 nt. PCR amplification was performed using 2XPrimeSTAR Mix (Takara, Japan). The target dsDNA fragments for the titration experiments were quantified using a PicoGreen dsDNA Quantitation Kit (Life iLab Biotech, China).

### DNA cleavage assays

Generally, *Pf*Ago-mediated cleavage assays were carried out in a reaction buffer (15 mM Tris/HCl pH 8.0, 250 mM NaCl, and 0.5 mM MnCl_2_). For ssDNA cleavage, 0.2 μM *Pf*Ago, 2 μM gDNA, and 0.8 μM ssDNA target were mixed in a reaction buffer and then incubated for 20 min at 95 °C in a thermocycler. Following high-temperature incubation, the samples were cooled by slowly lowering the temperature at a rate of 0.1 °C/s until they reached 10 °C. The reactions were stopped through the addition of loading buffer (95% formamide, 0.5 mM EDTA, 0.025% bromophenol blue, 0.025% xylene cyanol FF) at a 1:1 ratio (v/v), and then separated using 16% denaturing polyacrylamide gels and analyzed via staining with GelRed (Biotium, USA). The nucleic acids were visualized using a Tanon 2500 system (Shanghai, China) and quantitatively analyzed using ImageQuant (GE Healthcare, USA).

For the 600 bp dsDNA cleavage assays, 0.16 μM *Pf*Ago, 2 μM gDNAs, and 60 nM dsDNA target were mixed in a reaction buffer; for the 95 bp dsDNA cleavage assays, 0.2 μM *Pf*Ago, 1 μM gDNAs, and 180 nM dsDNA target were mixed in reaction buffer before incubation for 15 min at 95 °C in a thermocycler (Eppendorf, Germany). Following this high-temperature incubation, the samples were cooled by slowly lowering the temperature at a rate of 0.1 °C/s until it reached 10 °C. Reactions were quenched with 5 × DNA loading buffer (Generay, China) and analyzed on 2% agarose gels or 15% non-denaturing polyacrylamide gels. The gels were stained and analyzed as described above.

To investigate the 5ʹ-phosphorylated end of the cleavage fragment, 0.5 μM *Pf*Ago, 2 μM gDNAs, and 2 μM primary ssDNA target were mixed in reaction buffer. The mixture was incubated for 35 min at 95 °C, then the products of the primary ssDNA target cleavage mediated by primary gDNAs were treated with or without alkaline phosphatase during further incubation with secondary ssDNA targets for 35 min at 95 °C. The reactions were stopped via the addition of loading buffer (95% formamide, 0.5 mM EDTA, 0.025% bromophenol blue, 0.025% xylene cyanol FF) in a 1:1 ratio before the samples were resolved on 16% denaturing polyacrylamide gels. The gels were stained using GelRed (Biotium, USA), and nucleic acids were visualized using a Tanon 2500 system (Shanghai, China).

### Mass spectrometry analysis for the validation of the 5ʹ-terminal end of cleavage fragments

To validate the 5ʹ-terminal end of the cleavage fragment produced by gDNA-assisted *Pf*Ago activity, an in vitro cleavage reaction was performed using the ssDNA target cleaved by a single gDNA. The in vitro assay consisted of 0.5 μM *Pf*Ago, 2 μM single gDNA, and 2 μM ssDNA target in a total volume of 100 µL; for the control reactions, the gDNA was replaced with ultrapure water. The cleavage reactions were incubated at 95 °C for 1 h, and the reaction products were purified using an ethanol precipitation method in which a one-tenth volume of 3 M NaOAc and three volumes of ice-cold ethanol were added. The samples were kept at -20 °C for 2 h and subsequently centrifuged for 15 min at 12,000 rpm. The supernatant was removed, two volumes of 80% ethanol was added, and the pellet was incubated at − 20 °C for 2 h. The supernatant was decanted, and the samples were centrifuged for 5 min at 12,000 rpm. The pellet was air-dried, and the samples were resuspended in 50 μL of ultrapure water.

A Model 1290 Ultra Performance LC (UPLC) system (Agilent Technologies) with a Zorbax XDB C8 reversed-phase column (4.6 × 150 mm, 5 μm particle size, Agilent Technologies) was used for sample separation, with the column temperature controlled at 30 °C. The two mobile phase solvents consisted of buffer A (H_2_O with 0.1% ammonium hydroxide [v/v]) and buffer B (acetonitrile). The flow rate of the mobile phase was 0.4 mL/min and the injection volume was 5 μL. The LC flow was coupled to an Agilent model 6230 accurate-mass time-of-flight (TOF) mass spectrometer (MS) (Agilent Technologies) equipped with an Agilent Jet Stream electrospray ionization (ESI) source. The MS was operated in negative ion mode with the following parameters: capillary voltage, 3500 V; skimmer voltage, 65 V; nozzle voltage, 800 V; and fragmentor, 135 V. Nitrogen was used as the drying (8 L/min, 325 °C), sheath (11 L/min, 350 °C), and nebulizer gas (35 psi). The MS was tuned for large-MW ions, and data were acquired for m/z 400–5000. Data were saved in centroid mode using Agilent MassHunter Workstation Data Acquisition Software (revision B.04), and the MaxEnt deconvolution algorithm was used to generate a calculated neutral mass spectrum from the negatively charged ion data.

### Electrophoresis mobility shift assays

For electrophoresis mobility shift assays (EMSAs), 1 μM *Pf*Ago was pre-incubated with 0.2 μM fluorescently labeled gDNAs for 5 min at 95 °C in a reaction buffer (15 mM Tris/HCl pH 8.0, 250 mM NaCl, and 0.5 mM MnCl_2_. gDNAs without fluorescent labels were then added at various concentrations (0, 0.2, 0.4, 0.8, and 2.0 μM), and the reaction mixtures were incubated for an additional 5 min. Next, gel loading buffer (2.5% Ficoll 400, 62.5 mM Tris/HCl, pH 6.8) was added to quench the reaction, and the samples were analyzed using 8% non-denaturing polyacrylamide gels. Subsequently, the fluorescently labeled gDNA and the protein–gDNA complexes were visualized on a Fuji FLA7000 scanner with fluorescence measurements at *λ*_ex_ = 535 nm and *λ*_em_ = 595 nm.

### Fluorophore quencher (FQ)-labelled reporter assays

*Pf*Ago cleavage assays with FQ-labeled ssDNA were carried out in a reaction buffer (15 mM Tris–HCl, 250 mM NaCl, 0.5 mM MnCl_2_, pH 8.0). Using FQ-labeled ssDNA as an indicator of primary cleavage, the reactions were mixed to final concentrations of 300 nM *Pf*Ago, 2 µM gDNA, and 600 nM ssDNA-FQ reporter substrates in a total reaction volume of 25 µL. To measure the effect of gDNA concentration on secondary cleavage efficiency, the reactions were mixed to final concentrations of 300 nM *Pf*Ago, 800 nM target ssDNA, and 600 nM ssDNA-FQ reporter substrates in a 25-µL reaction volume, and the gDNA was added separately to final concentrations of 0, 0.4, 0.8, 1.2, 1.6, 2.0, 4.0, and 8.0 µM. To measure the effect of target ssDNA concentration on secondary cleavage efficiency, the reactions were mixed to final concentrations of 300 nM *Pf*Ago, 800 nM gDNA, and 600 nM ssDNA-FQ reporter, and the target ssDNA was added separately to final concentrations of 0, 40, 80, 200, 320, 400, 600, and 800 nM. To measure the effect of the *Pf*Ago concentration on secondary cleavage efficiency, the reactions were mixed to final concentrations of 800 nM target ssDNA, 800 nM gDNA, and 600 nM ssDNA-FQ reporter, and *Pf*Ago was added separately to final concentrations of 0, 40, 80, 200, 320, 400, 600, and 800 nM. Reactions were incubated in a Mastercycler® RealPlex instrument (Eppendorf, Germany) for up to 30 min at 95 °C.

For multiplex detection of ssDNA targets, reactions were performed as follows. The components of the reaction were 1.2 µM *Pf*Ago, 0.4 µM gDNA for each DNA, 0.4 µM target ssDNA for each DNA, and the ssDNA-FQ reporter was added as 0.4 µM FAM-labeled ssDNA-FQ reporter, 0.8 µM JOE-labeled ssDNA-FQ reporter, 1.2 µM NED-labeled ssDNA-FQ reporter, or 2 µM ROX-labeled ssDNA-FQ reporter. Reactions (25 µL, Axygen qPCR tube) were incubated in a Mastercycler® RealPlex instrument (Eppendorf, Germany) for up to 30 min at 95 °C, with fluorescence measurements taken every 30 s (FAM-labeled ssDNA-FQ reporter *λ*_ex_: 495 nm, *λ*_em_: 520 nm; JOE-labeled ssDNA-FQ reporter *λ*_ex_: 529 nm, *λ*_em_: 550 nm; NED-labeled ssDNA-FQ reporter *λ*_ex_: 557 nm, *λ*_em_: 580 nm; ROX-labeled ssDNA-FQ reporter *λ*_ex_: 586 nm, *λ*_em_: 605 nm).

For dsDNA secondary cleavage analysis, dsDNAs were prepared from HPV-16-containing plasmids using PCR. Briefly, 25 µL reactions containing 0.5 µL of plasmid (290 ng/µL), 0.6 µM forward and reverse primers, and 2 × AceQ qPCR Probe Master Mix (Vazyme Biotech, China) were used to amplify the target dsDNA fragments. The dsDNA product (2 pM) was mixed with 0.4 µM FAM-labelled ssDNA-FQ reporter, 1.2 µM *Pf*Ago, 0.5 mM MnCl_2_ and 2 µM reverse gDNA (targeting the secondary gDNA production chain) or both reverse gDNA and forward gDNA. Reactions were incubated in a Mastercycler® RealPlex instrument (Eppendorf, Germany) for up to 30 min at 95 °C, and fluorescence measurements were taken every 30 s (ssDNA-FQ reporter *λ*_ex_: 495 nm, *λ*_em_: 520 nm).

For Michaelis–Menten analysis, 0.8 μM *Pf*Ago and 0.8 μM gDNA were mixed in a reaction buffer, and the reactions were initiated by adding 0.04, 0.08, 0.2, 0.4, 0.6, or 0.8 µM substrate (FQ-labelled ssDNA). The reactions were incubated in a Mastercycler® RealPlex instrument (Eppendorf, Germany) for up to 990 s at 95 °C, and fluorescence measurements were taken every 10 s (*λ*_ex_: 535 nm, *λ*_em_: 595 nm).

### Multiple ssDNA detection

A four-channel multiplex ssDNA detection system was established for four different types of target genes in a one-tube reaction system. The reaction system was similar to the aforementioned FQ-labeled reporter assays, with the following modifications: 25 µL reactions contained 400 nM target ssDNA (*PIK3CA*, *KRAS*, *NRAS*, and *EGFR*), 0.4 µM gDNAs (ssDNA1, ssDNA2, ssDNA3, ssDNA4 targeting), 2.4 µM *Pf*Ago, 0.4 µM FAM-labeled ssDNA-FQ reporter (ssDNA1), 0.8 µM JOE-labeled ssDNA-FQ reporter (ssDNA2), 1.2 µM NED-labeled ssDNA-FQ reporter (ssDNA3), and 2 µM ROX-labeled ssDNA-FQ reporter (ssDNA4). Reactions were incubated in a Mastercycler® RealPlex instrument (Eppendorf, Germany) for up to 30 min at 95 °C with fluorescence measurements taken every 30 s (FAM-labeled ssDNA-FQ reporter *λ*_ex_: 495 nm, *λ*_em_: 520 nm; JOE-labeled ssDNA-FQ reporter *λ*_ex_: 529 nm, *λ*_em_: 550 nm; NED-labeled ssDNA-FQ reporter *λ*_ex_: 557 nm, *λ*_em_: 580 nm; ROX-labeled ssDNA-FQ reporter *λ*_ex_: 586 nm, *λ*_em_: 605 nm).

### RADAR assays

HPV detection assays were performed as described above with the following modifications: 50 µL pre-amplification reactions contained 0.5 µL of plasmid for each gene (40 ng/µL) and 0.5 µM universal primers. Then, HPV-6-, HPV-11-, HPV-16-, and HPV-18-targeting gDNAs, at 0.4 µM concentration of each gDNA, were added in each reaction tube; 2.4 µM *Pf*Ago; 0.4 µM FAM-labeled ssDNA-FQ reporter (HPV-16); 0.8 µM JOE-labeled ssDNA-FQ reporter (HPV-18); 1.2 µM NED-labeled ssDNA-FQ reporter (HPV-6); and 2 µM ROX-labeled ssDNA-FQ reporter (HPV-11) were added to the PCR mixtures. Fluorescence detection reactions (35 µL final volume) were carried out in 0.2 mL PCR strip tubes (Axygen, USA) and incubated in a Mastercycler® RealPlex instrument (Eppendorf, Germany). Initially, 30 cycles of PCR amplification were conducted (95 °C, 30 s; 60 °C, 20 s), then the samples were incubated for 30 min at 95 °C with fluorescence measurements taken every 30 s (FAM-labeled ssDNA-FQ reporter *λ*_ex_: 495 nm, *λ*_em_: 520 nm; JOE-labeled ssDNA-FQ reporter *λ*_ex_: 529 nm, *λ*_em_: 550 nm; NED-labeled ssDNA-FQ reporter *λ*_ex_: 557 nm, *λ*_em_: 580 nm; ROX-labeled ssDNA-FQ reporter *λ*_ex_: 586 nm, *λ*_em_: 605 nm).

To evaluate the detection sensitivity of RADAR, the HPV-16 plasmid template was diluted to 10^–10^, 10^–11^, 10^–12^, 10^–13^, 10^–14^, 10^–15^, 10^–16^, 10^–17^, 10^–18^, 10^–19^, and 10^–20^ M. With the same detection system, the template was analyzed separately at these concentrations, and the detection values were normalized to the maximum mean fluorescence signal. For HPV clinical sample identification using RADAR, the detection method was the same as above, with the detection of HPV-16 or HPV-18 in human samples implemented using *Pf*Ago targeting the hypervariable loop V of the *L1* gene within HPV-16 or HPV-18. One-way ANOVA with Dunnett’s post-test was used to determine the positive cutoff (set at *p* ≤ 0.05) for the identification of HPV-16 or HPV-18 in patient samples.

### Human clinical sample collection and DNA preparation and validation

Donors providing anal swab samples were recruited from the Fifth People’s Hospital of Shanghai, Fudan University (Shanghai, China). This study was approved by the Human Research Committee of the Fifth People’s Hospital of Shanghai, Fudan University (Shanghai, China). The samples were collected from an anal swab into a Thinprep™ vial with 1 mL of sterile saline for HPV testing. The cell suspension was centrifuged for 5 min at 12,000 rpm, and the cell precipitate was resuspended in 1 mL of sterile saline used for DNA extraction with a TIANamp Genomic DNA kit (TianGen Biotechnologies, Beijing). Subsequently, 5 μL of DNA was used for HPV consensus PCR analysis and the RADAR test in parallel.

PCR-based HPV genotyping and validation were performed using a commercially available Liferiver HPV Genotyping Real Time PCR Kit (ZJ Bio-Tech Corporation, Shanghai). This method is based on the use of sequence-specific probes for real-time TaqMan detection. After 40 amplification cycles, the specimens were probed using a FAM-labeled specific probe mixture. Specimens negative for beta-globin gene amplification were excluded from the analysis. The results were based on the Ct value (< 38) and amplification curve (S-shape) of real-time PCR.

## Results and discussion

### Elucidation of the stepwise endonuclease activity of PfAgo

**Fig. 1:**
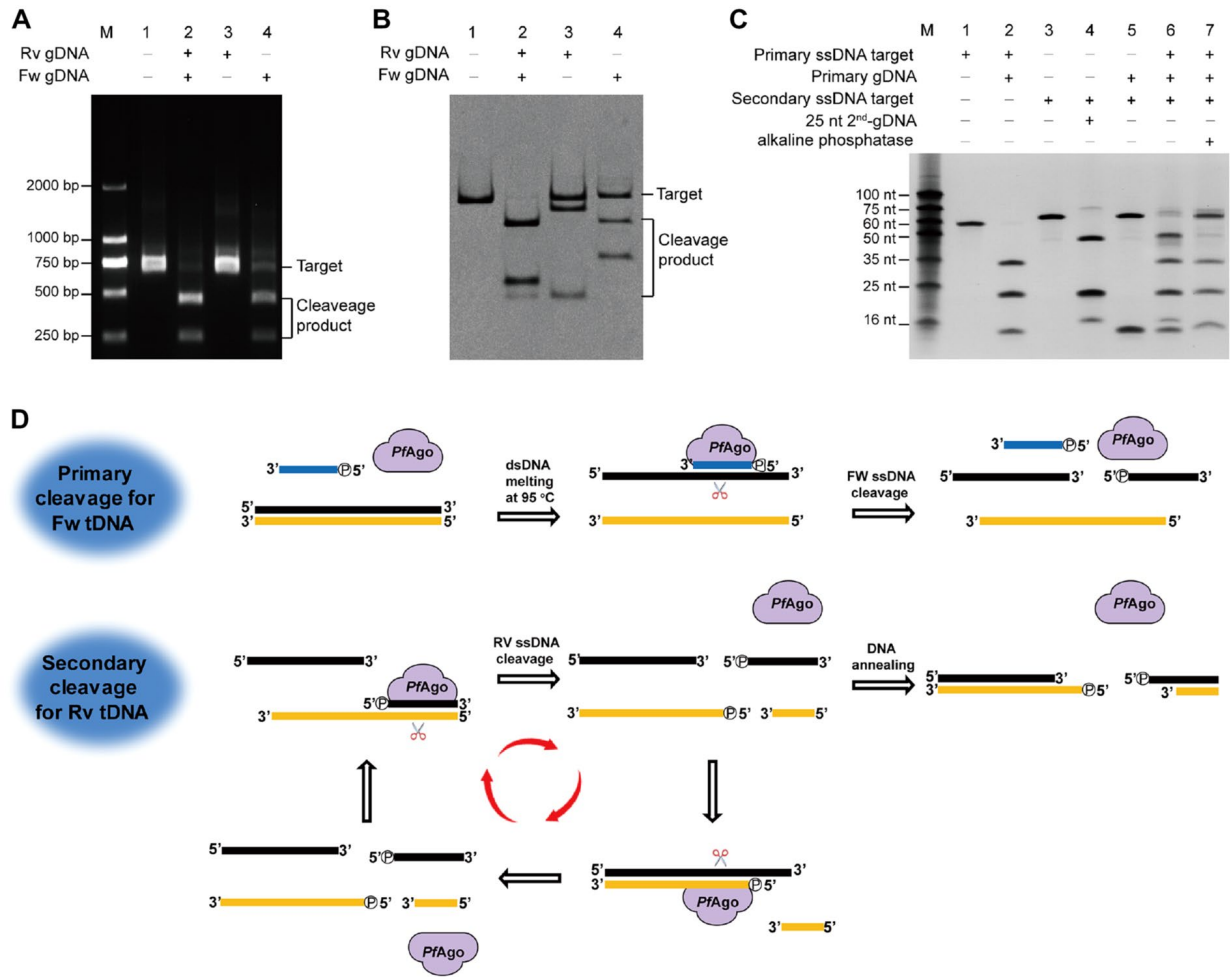
*Pf*Ago-targeted DNA cleavage mediated by a single gDNA. **A** A single gDNA can mediate *Pf*Ago activity for 600-nucleotide (bp) long DNA targets to produce DNA cleavage products. **B** A single gDNA can assist *Pf*Ago-mediated cleavage of 95-bp DNA targets, producing distinct banding patterns from reactions containing a pair of gDNAs. **C** A cleavage fragment with a 5′-phosphate end can function as a renewed gDNA that can initialize an additional round of DNA cleavage if the reverse complement sequence of the original target is present. **D** Schematic of single gDNA-assisted *Pf*Ago-mediated activity for targeted DNA cleavage

We were initially interested in characterizing the cleavage performance of the hyperthermostable *Pf*Ago at 95 °C in vitro, in which gDNAs were designed to target a 600-bp PCR fragment as the cleavage template sequence (Additional file 1: Table S1, Figure S1). In previous reports of hyperthermophilic *Pf*Ago, a single gDNA directly cleaves ssDNA target. Thus, a pair of gDNAs is designed to break each stand of dsDNA target in two independent processes. Unexpectedly, we found that a single gDNA could mediate *Pf*Ago activity to cleave both strand of dsDNA rather than a nick pattern (Fig. 1A). When using a short PCR fragment as the target template (95 bp), *Pf*Ago was also able to cleave DNA in the presence of a single gDNA. Surprisingly, distinct banding patterns were observed for the cleavage products in reactions that used a single gDNA or a pair of gDNAs (Fig. 1B). These observations suggest that there may be some unusual enzymatic reaction processes involved in this cleavage.

For this apparently off-target cleavage phenomenon, we first successfully confirmed that *Pf*Ago was able to bind single gDNAs and undergo a guide-replacement process (Additional file 1: Figure S2). We then conducted mass spectrometry analysis of products from *Pf*Ago-mediated reactions that used a single gDNA to cleave a 60-nt ssDNA target. We detected two products from this reaction: a 25-nt product with a 5'-phosphate end and a 35-nt product with a 3'-hydroxyl end (Additional file 1: Figure S3). Given that 5'-phosphate ends are required for the activity of gDNAs in facilitating *Pf*Ago-mediated cleavage, our detection of cleaved ssDNA fragments with the 5ʹ-phosphate ends raised the possibility that such fragments could potentially function as a “renewed gDNA” that can be used for further rounds of stepwise cleavage to the reverse complement sequences.

To probe the function of the above cleavage products of the 60-nt ssDNA target, we added the reverse complement 60-nt ssDNA to the reaction mixture from the primary reaction. We then detected the expected 15- and 45-nt cleavage products as the cleavage of reverse complement ssDNA (Fig. 1C). Moreover, we found that enzymatic dephosphorylation treatment of the cleavage products from the primary reaction completely abolished any stepwise secondary cleavage activity for the reverse complement ssDNA. These results together support our hypothesis that cleavage fragments with 5ʹ-phosphate ends generated in a primary cleavage can be used as “renewed gDNA” for stepwise rounds of cleavage that target the reverse complement DNA sequence. Given that such subsequent rounds would also generate additional products with 5ʹ-phosphate ends, we envisioned a cascade of DNA cleavage rounds that targeted the two complementary sequences with each of the corresponding newly generated gDNAs (Fig. 1D). In the in vitro guide-free assays of *Tt*Ago and *Mj*Ago, autonomous guide generation resulted from small dsDNA fragments could degrade dsDNA in a “chopping” phenomenon (Swarts et al. 2017; Zander et al. 2017). Our results demonstrated that the stepwise activity of *Pf*Ago is supported by a single gDNA, which clearly elucidates the role of the guide in the catalytic process by a renewed guide mechanism.

### Development of RADAR for DNA detection

**Scheme 1:**
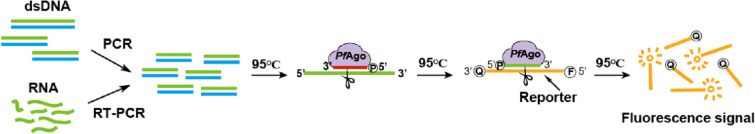
Design of RADAR for target nucleic acid detection

Because *Pf*Ago enabled precise cleavage via short gDNA that can be designed arbitrarily to any target sequence, we anticipated *Pf*Ago-based diagnostics to offer a unique opportunity for developing virus detection methods. Based on *Pf*Ago’s stepwise cleavage, we exploited it as a target DNA detection platform comprising the initial ssDNA templates, a designed gDNA targeting the template, *Pf*Ago, and reverse complement FQ-ssDNA reporter substrates. In this design, the gDNA assists *Pf*Ago-mediated cleavage of the ssDNA template in the primary reaction, thereby generating cleavage products to subsequently assist in the secondary cleavage of the FQ-ssDNA reporter substrates. We tested this concept, termed RADAR (Renewed-gDNA Assisted DNA cleavage with Argonaute, Scheme 1). Compared to CRISPR diagnostics that require long RNAs as guides (Chen et al. 2018b; Gootenberg et al. 2018; Li et al. 2018), our guide design used a low-cost, highly stable short gDNA, allowing for more broad in vitro assays.

**Fig. 2:**
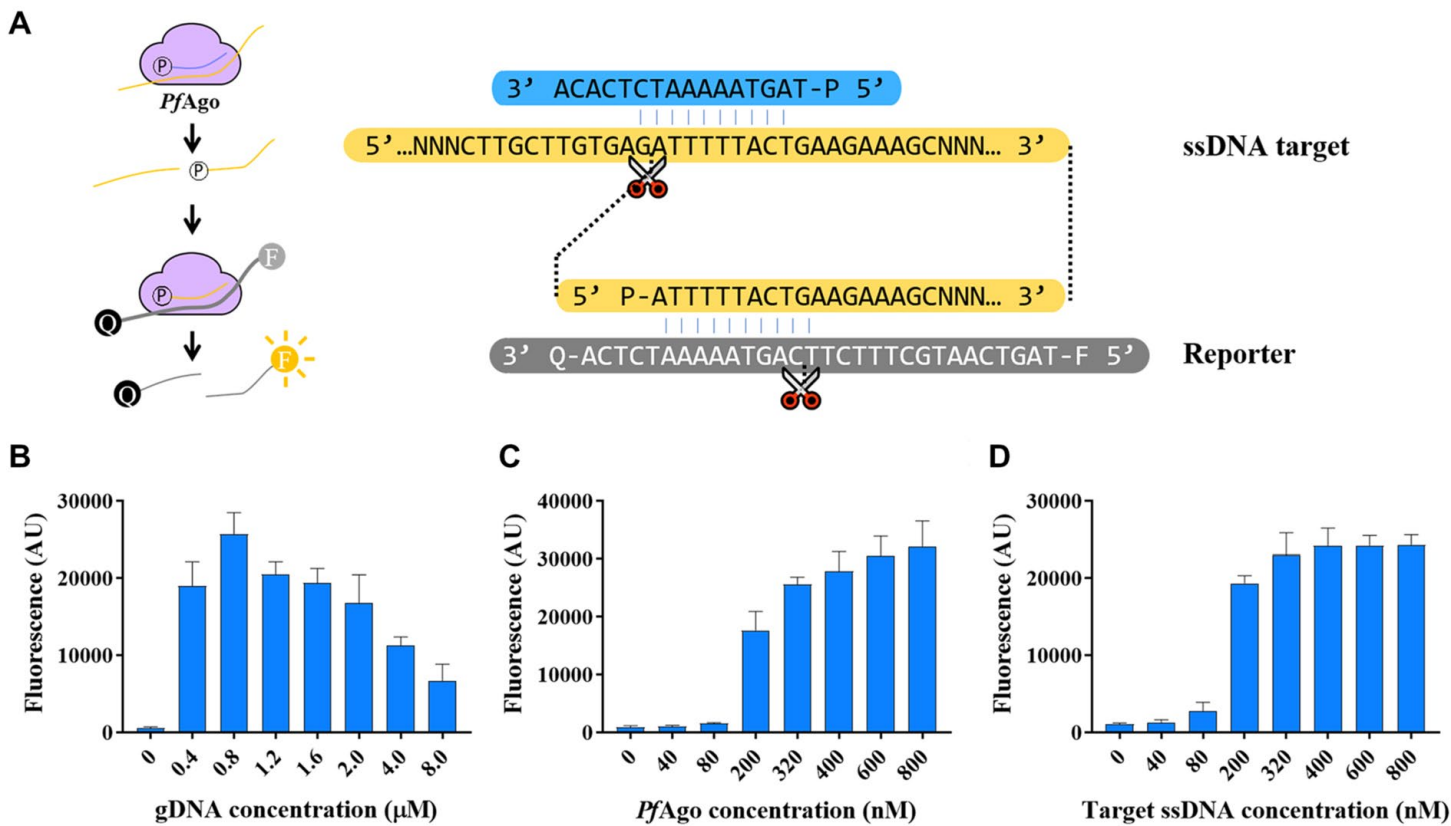
Optimization of RADAR detection. **A** Schematic of assay to determine single gDNA-assisted *Pf*Ago-mediated cleavage activity. **B** Single gDNA-assisted *Pf*Ago-mediated cleavage activity with gDNAs of varying concentration when the concentration of *Pf*Ago is 0.3 µM and that of the target ssDNA is 0.8 µM. **C** Single gDNA-assisted *Pf*Ago-mediated cleavage activity of *Pf*Ago with ssDNA of varying concentration when the concentration of *Pf*Ago is 0.3 µM and that of the gDNA is 0.8 µM. **D** Single gDNA-assisted *Pf*Ago-mediated cleavage activity of *Pf*Ago with *Pf*Ago of varying concentration when the concentration of gDNA is 0.8 µM and that of the target ssDNA is 0.8 µM. Each bar represents the mean fluorescence signal from the fluorophore-labeled ssDNA cleavage after 40 min at 95 °C. Error bars represent the mean ± s.d., where *n* = 3 replicates

We then systematically optimized the conditions in the single gDNA-assisted cleavage system, including the ratios of gDNA:*Pf*Ago:target DNA (Fig. 2A–D), and the gDNA tiling reporter length (Additional file 1: Figure S4). Next, we conducted the system based on single gDNA-assisted *Pf*Ago-mediated DNA cleavage to probe target ssDNA, in which gDNA (16 nt), ratios of *Pf*Ago:gDNA:target DNA (3:8:8), along with the gDNA tiling reporter (30 nt). We also investigated the kinetics of single gDNA-assisted *Pf*Ago-mediated cleavage using 30-nt ssDNA substrates labeled with fluorophores at their 5ʹ-ends and fluorophore-quenchers at their 3'-ends (FQ-ssDNA). The single gDNA-assisted *Pf*Ago-mediated cleavage activity, monitored quantitatively via fluorescence release, fits the Michaelis–Menten equation with a catalytic efficiency (*k*_cat_/*K*_m_) of 7.1 × 10^7^ s^−1^ M^−1^ (Additional file 1: Figure S5). In PAND detection, one to three input gDNAs were tested, and three guide DNAs with 10 pM *Pf*Ago and 0.3 pM gDNA can significantly obtain higher signals than those when using one or two guides (He et al. 2019). Here, after systematic optimization of RADAR, only one gDNA is needed to activate the cyclic cleavage, which is simpler and more efficient for further design and operation. We reasoned that the ratio of *Pf*Ago: gDNA was the key factor for achieving the best performance for target detection.

### Specificity and sensitivity of RADAR

**Fig. 3:**
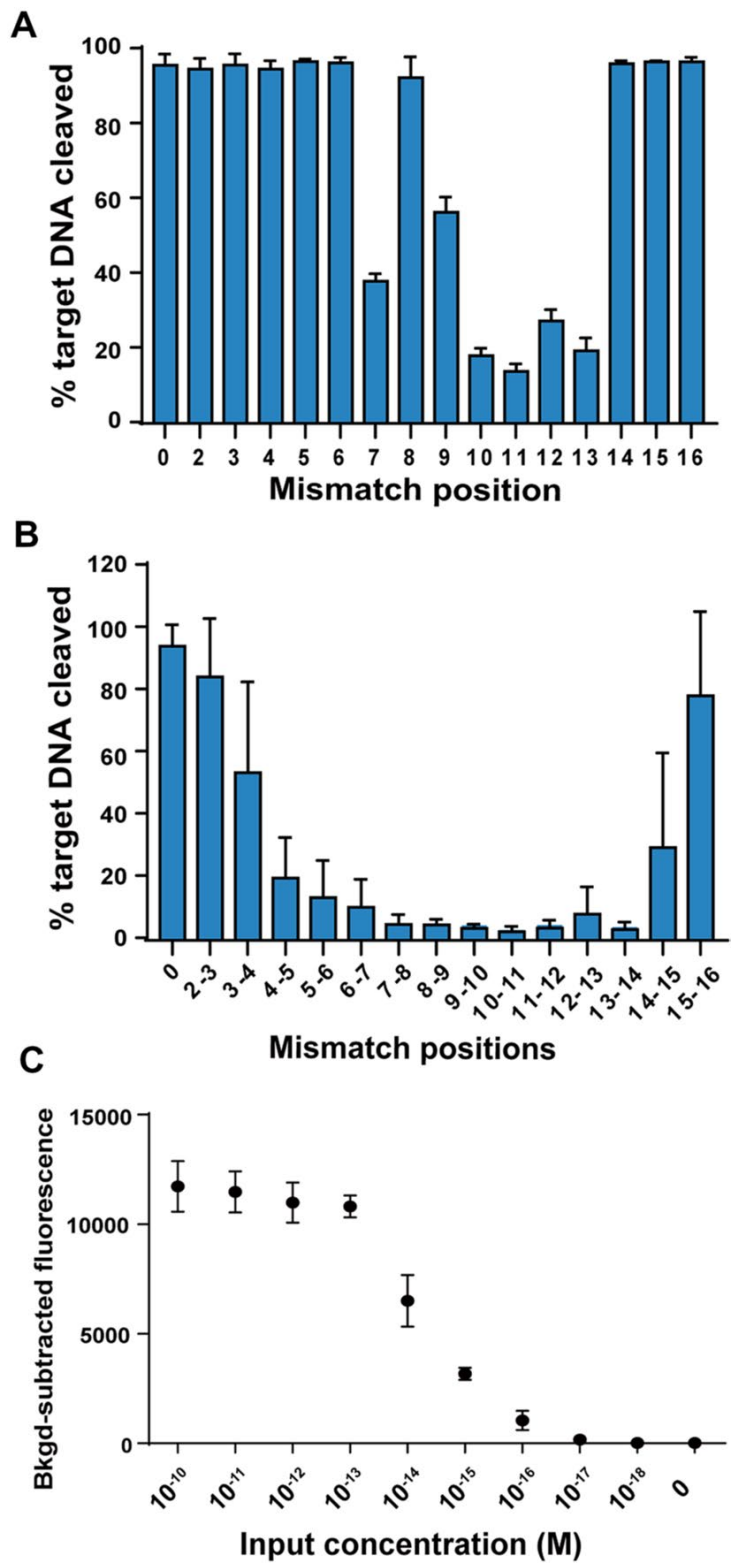
The specificity and sensitivity of RADAR detection. Observed activity for *Pf*Ago using a fluorophore-labeled ssDNA with the indicated single nucleotide mismatches (**A**) and di-nucleotide mismatches (**B**). *Pf*Ago cleavage kinetics of ssDNA targets were measured, and the cleavage activity at the 20 min time point was plotted against the mismatch position. Ratios represent the average of three different targets measured in triplicate, and error bars represent mean ± s.d., where *n* = 9 (three replicates for three independent targets. **C** Detection sensitivity of the RADAR system using diluted plasmids. Error bars represent the mean ± s.d., *n* = 3

To test the on-target cleavage specificity of target ssDNA, we designed multiple gDNAs targeting ssDNA and included mismatches across the length of the gDNA. These experiments showed that positions 10–13 of the gDNA sequence confer target specificity (Fig. 3A, B, Additional file 1: Figure S6), which is similar to the mismatch tolerance pattern indicated by *Tt*Ago (Wang et al. 2008). Moreover, these assays showed that di-nucleotide mismatches between gDNA and target sequences significantly decreased the cleavage activity of *Pf*Ago. These results suggest the feasibility of programming the cleavage specificity of Ago to enable discrimination among target DNAs that differ by as little as two-nucleotide mismatches. Furthermore, due to the low absolute target concentration within the genomic background, clinical samples are usually treated with a pre-amplification step. To improve the detection sensitivity of RADAR, we added PCR amplification to target sequences prior to subsequent *Pf*Ago-based detection. The diluted plasmids were used to determine detection sensitivity, which were as low as the femtomolar level (Fig. 3C). With the aid of the highly specific cleavage and programmable ability, a high sensitivity and specificity was achieved by the precise cleavage of both primary cleavage to variant tDNA and secondary cleavage to the specially designed reporter readout system. Furthermore, we proposed that RADAR can be applied to enable a simple and fast interpretation of the variant sharing of results directly with the convenience and easy handling as a single tube reaction.

### Feasibility of multiplex detection

**Fig. 4:**
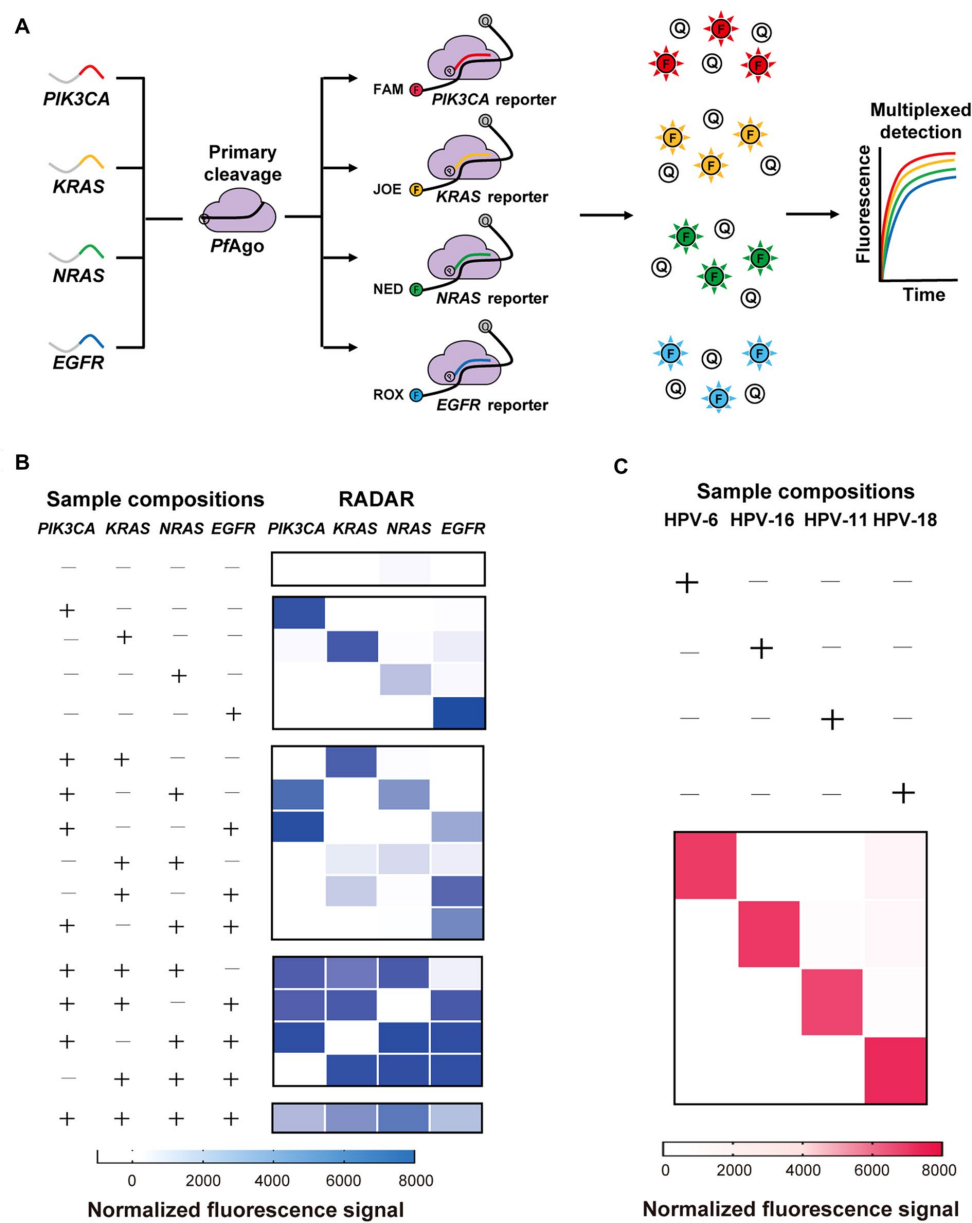
RADAR for multiplex detection of nucleic acids. **A** Schematic of one-tube four-fluorophore multiplexing using orthogonal single-gDNA-assisted *Pf*Ago-mediated cleavage activity, with each gDNA designed for respective ssDNA targets. **B** In-sample multiplexed detection of four ssDNA targets with corresponding gDNAs designed in an orthogonal manner. **C** Multiplexed detection of four HPV dsDNA targets with corresponding gDNAs designed in an orthogonal manner

Because of the orthogonal guide-directed specific cleavage of *Pf*Ago, we extended RADAR to test its ability to simultaneously discriminate among multiple target sequences. We tested four synthesized ssDNA templates (each 60 nt long) based on the sequences of four different oncogenes (*PIK3CA*, *KRAS*, *NRAS*, and *EGFR*) (Fig. 4A). For multiplex detection, we used a *PIK3CA*-FAM reporter, a *KRAS*-JOE reporter, a *NRAS*-NED reporter, and an *EGFR*-ROX reporter, and we were able to detect each of the four targets in a single reaction (Fig. 4B, Additional file 1: Figure S7). Notably, no off-target signals were found in any of the reaction combinations, and the intensities of each of the four output signals in the multiplex reaction were at similar levels.

**Fig. 5:**
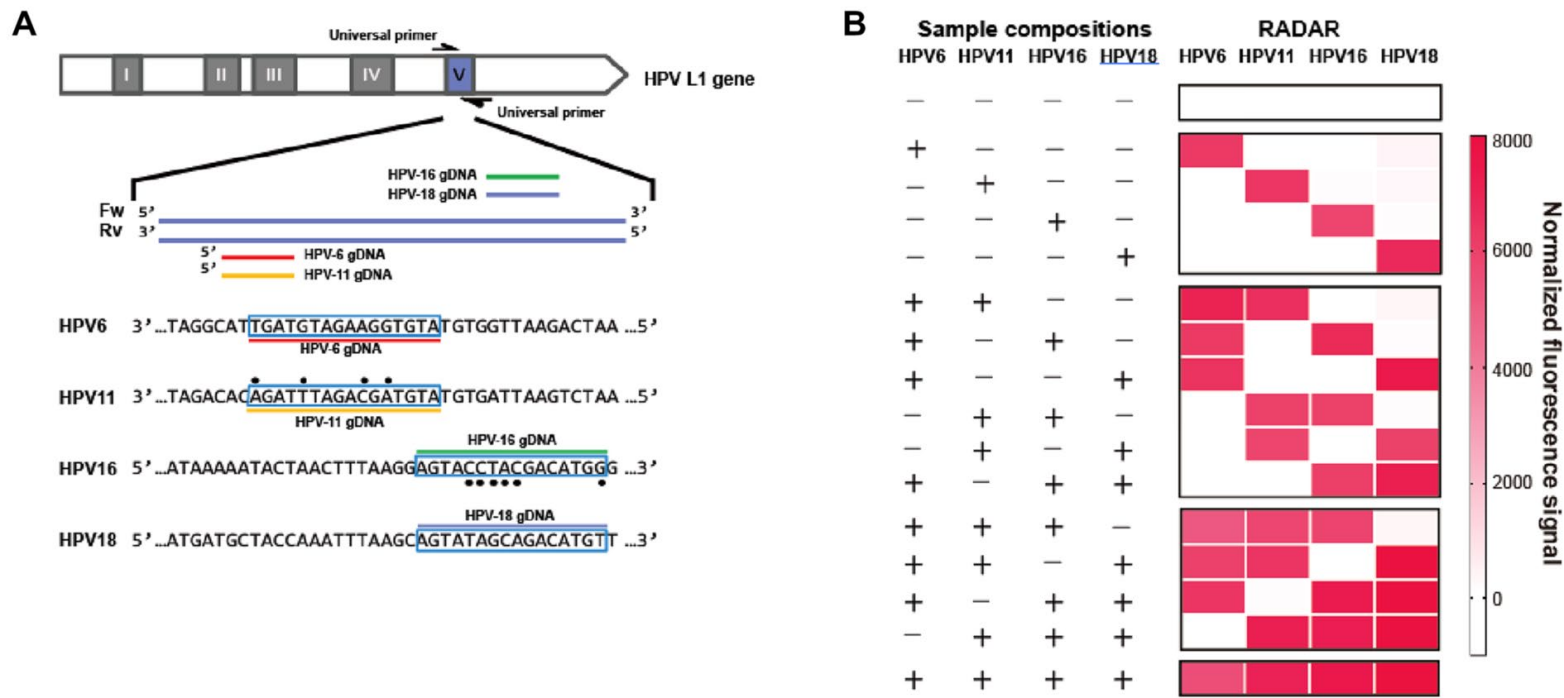
Multiplex RADAR detection applied for HPV genotyping. **A** Sequences of the four HPV serotypes within the hypervariable loop V of the *L1* gene targeted by gDNAs and *Pf*Ago. The highlighted bases indicate the designed gDNA sequence. **B** RADAR detection of one-tube multiplex detection of four dsDNA targets of HPV serotypes with the corresponding designed gDNAs

We then detected the presence of the four most common serotypes of human papillomavirus (HPV): types 6, 11, 16, and 18 (Fig. 4C) (Woodman et al. 2007). First, we prepared samples containing various combinations of purified plasmids harboring serotype-specific alleles of the viral HPV *L1* gene. These samples were used as templates for the amplification of approximately 110 bp amplicons using universal HPV primers. Subsequently, the amplified samples were reacted in a one-tube system containing four serotype-specific gDNAs and four serotype-specific FQ-ssDNA reporters (Fig. 5A). By establishing a combination of a PCR amplification step and our one-tube multiplexed detection via orthogonal cleavage by *Pf*Ago, these RADAR experiments correctly and unambiguously identified each of the HPV subtypes present in each of the sample mixtures (Fig. 5B, Additional file 1: Figure S8). Thus, we established a proof-of-concept for the one-tube, multiplexed detection of multiple DNA substrates via orthogonal cleavage by *Pf*Ago. In contrast, CRISPR-based multiplexing relies on the non-specific cleavage of the same type of nucleotide being sensed, and different Cas effector proteins with different nucleotide preferences are needed for multiple targets, which require enzyme screening and increase system complexity (Gootenberg et al. 2018).

### Validation of clinic samples for HPV genotyping

To apply RADAR to samples from human patients, we tested DNA samples extracted from human anal swabs that had been previously analyzed using a PCR-based method for HPV infection (Additional file 1: Figure S9). Within 2 h, our RADAR method accurately identified HPV-16 (9/9 agreement) and HPV-18 (7/7 agreement) infections in clinical samples, with good correlation between the PCR-based intensity and RADAR signals (Additional file 1: Figure S10). We demonstrate that our *PfAgo-*cleavage-based RADAR can be used as a fast, sensitive, and reliable platform for the multiplexed detection of viral DNA in human samples.

## Conclusions

In summary, our study revealed the stepwise endonuclease activity of thermophilic *Pf*Ago, which may provide a reprogrammable, efficient, and precise route for DNA editing. Our biochemical analysis demonstrated that the single primary gDNA-directed dsDNA cleavage is the result of cleavage via precise generation of a “renewed gDNA guide”. Through the stepwise cleavage and orthogonal target specificity of *Pf*Ago, we successfully established a one-tube multiplexed DNA detection platform, known as RADAR, for genotyping. Here, we anticipated RADAR as a versatile and sensitive method in molecular diagnosis, especially for its advantage in cheap and accessible gDNA design, arbitrary cleaved target sequences, and operational simplicity. The exploration of thermophilic endonucleases is an alternative option for DNA editing, thus leading to a range of applications in medical diagnosis.

### Supplementary Information

**Additional file 1: Figure S1.** Purification of *Pf*Ago protein. **Figure S2.** Detection dynamic process of *Pf*Ago binding gDNA using an electrophoresis mobility shift assay (EMSA). **Figure S3.** TOF–MS analysis of cleavage ends from reaction of *Pf*Ago with ssDNA target. **Figure S4.** Testing single gDNA-assisted *Pf*Ago-mediated cleavage activity effected by gDNAs tiling reporter. **Figure S5.** Michaelis–Menten analysis reveals single gDNA-assisted *Pf*Ago-mediated cleavage activity with a ssDNA-FQ reporter. **Figure S6.** The proximal mismatches of gDNA with dsDNA target in the assay of fluorescent reporter provide specificity for single gDNA-assisted *Pf*Ago-mediated cleavage activity. **Figure S7.** Multiplex detection based on orthogonal single gDNA-assisted *Pf*Ago-mediated cleavage activity with ssDNA targets. **Figure S8.** Multiplex detection of 4 subtypes HPV containing plasmids by RADAR. **Figure S9.** Identification of HPV serotypes by PCR based (left) and RADAR detection (right). **Figure S10.** RADAR analysis of HPV types 16 and 18 in patient samples. **Table S1.** *Pf*Ago protein used in this study. **Table S2.** gDNA used in this study. **Table S3.** ssDNA targets used in this study. **Table S4.** Primers used in this study. **Table S5.** Cleavage reporters used in this study. **Table S6.** Plasmids used in this study. **Table S7.** Nucleic acid for EMAS used in this study.

## Data Availability

All data generated or analyzed during this study are included in this article.

## Abbreviations

Ago: Argonaute protein
RADAR: Renewed-gDNA Assisted DNA-cleavage by Argonaute
ssDNA: Single strand DNA
gDNA: Guide DNA
dsDNA: Double strand DNA
RPA: Recombinase polymerase amplification
SNP: Single nucleotide polymorphism
PAM: Protospacer adjacent motif
PFS: Protospacer flanking site
NAVIGATER: Nucleic Acid enrichment Via DNA Guided Argonaute from *Thermus thermophilus*
PAND: *Pf*Ago-mediated Nucleic acid Detection
IPTG: Isopropyl β-d-thiogalactopyranoside
FQ: Fluorophore quencher
TOF: Time-of-flight
MS: Mass spectrometer
ESI: Electrospray ionization
EMSA: Electrophoresis mobility shift assay
